# Sanitation and Health

**DOI:** 10.1371/journal.pmed.1000363

**Published:** 2010-11-16

**Authors:** Duncan Mara, Jon Lane, Beth Scott, David Trouba

**Affiliations:** 1 School of Civil Engineering, University of Leeds, Leeds, United Kingdom; 2 Water Supply and Sanitation Collaborative Council, Geneva, Switzerland; 3 Department of Infectious and Tropical Diseases, London School of Hygiene and Tropical Medicine, London, United Kingdom

## Abstract

As one article in a four-part *PLoS Medicine* series on water and sanitation, David Trouba and colleagues discuss the importance of improved sanitation to health and the role that the health sector can play in its advocacy.

**Summary Points**

- 2.6 billion people in the world lack adequate sanitation—the safe disposal of human excreta. Lack of sanitation contributes to about 10% of the global disease burden, causing mainly diarrhoeal diseases.
- In the past, government agencies have typically built sanitation infrastructure, but sanitation professionals are now concentrating on helping people to improve their own sanitation and to change their behaviour.
- Improved sanitation has significant impacts not only on health, but on social and economic development, particularly in developing countries.
- The health sector has a strong role to play in improving sanitation in developing countries through policy development and the implementation of sanitation programmes.

**This is one article in a four-part** ***PLoS Medicine*** **series on water and sanitation.**

## Introduction and Definitions

Adequate sanitation, together with good hygiene and safe water, are fundamental to good health and to social and economic development. That is why, in 2008, the Prime Minister of India quoted Mahatma Gandhi who said in 1923, “sanitation is more important than independence” [1]. Improvements in one or more of these three components of good health can substantially reduce the rates of morbidity and the severity of various diseases and improve the quality of life of huge numbers of people, particularly children, in developing countries [2],[3]. Although linked, and often mutually supporting, these three components have different public health characteristics. This paper focuses on sanitation. It seeks to present the latest evidence on the provision of adequate sanitation, to analyse why more progress has not been made, and to suggest strategies to improve the impact of sanitation, highlighting the role of the health sector. It also seeks to show that sanitation work to improve health, once considered the exclusive domain of engineers, now requires the involvement of social scientists, behaviour change experts, health professionals, and, vitally, individual people.

Throughout this paper, we define sanitation as the safe disposal of human excreta [4]. The phrase “safe disposal” implies not only that people must excrete hygienically but also that their excreta must be contained or treated to avoid adversely affecting their health or that of other people.

## Health Impacts of Sanitation

Lack of sanitation leads to disease, as was first noted scientifically in 1842 in Chadwick's seminal “Report on an inquiry into the sanitary condition of the labouring population of Great Britain” [5]. A less scientifically rigorous but nonetheless professionally significant indicator of the impact on health of poor sanitation was provided in 2007, when readers of the BMJ (British Medical Journal) voted sanitation the most important *medical* milestone since 1840 [6].

The diseases associated with poor sanitation are particularly correlated with poverty and infancy and alone account for about 10% of the global burden of disease [7]. At any given time close to half of the urban populations of Africa, Asia, and Latin America have a disease associated with poor sanitation, hygiene, and water [8].

**Figure 1:**
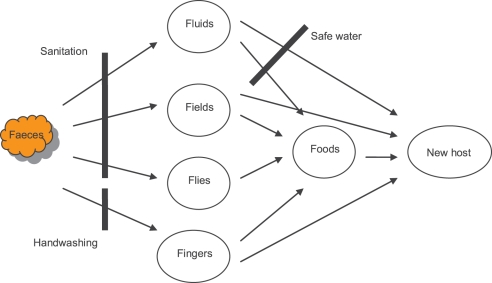
Faeco-oral disease transmission pathways and interventions to break them. Source: [10].

Of human excreta, faeces are the most dangerous to health. One gram of fresh faeces from an infected person can contain around 10^6^ viral pathogens, 10^6^–10^8^ bacterial pathogens, 10^4^ protozoan cysts or oocysts, and 10–10^4^ helminth eggs [9]. The major faeco-oral disease transmission pathways are demonstrated in the “F Diagram” (Figure 1) [10], which illustrates the importance of particular interventions, notably the safe disposal of faeces, in preventing disease transmission.

### Diarrhoeal Diseases

Diarrhoeal diseases are the most important of the faeco-oral diseases globally, causing around 1.6–2.5 million deaths annually, many of them among children under 5 years old living in developing countries [11],[12]. In 2008, for example, diarrhoea was the leading cause of death among children under 5 years in sub-Saharan Africa, resulting in 19% of all deaths in this age group [13].

Systematic reviews suggest that improved sanitation can reduce rates of diarrhoeal diseases by 32%–37% [14]–[16]. While many of the studies included in those reviews could not rigorously disaggregate the specific effects of sanitation from the overall effects of wider water, sanitation, and hygiene interventions, a longitudinal cohort study in Salvador, Brazil, found that an increase in sewerage coverage from 26% to 80% of the target population resulted in a 22% reduction of diarrhoea prevalence in children under 3 years of age; in those areas where the baseline diarrhoea prevalence had been highest and safe sanitation coverage lowest, the prevalence rate fell by 43% [17]. Similarly, a recent meta-analysis that explored the impact of the provision of sewerage on diarrhoea prevalence reported a pooled estimate of a 30% reduction in diarrhoea prevalence and up to 60% reduction in areas with especially poor baseline sanitation conditions [18]. Another longitudinal study in urban Brazil found that the major risk factors for diarrhoea in the first three years of life were low socioeconomic status, poor sanitation conditions, presence of intestinal parasites, and absence of prenatal examination. The study concluded that diarrhoeal disease rates could be substantially decreased by interventions designed to improve the sanitary and general living conditions of households [19].

Further, it is not just the provision and adult use of sanitation that is important. A meta-analysis of observational studies of infants' faeces disposal practices found that unsafe disposal increased the risk of diarrhoea by 23%, highlighting the importance of the safe management of both adults' and infants' faeces [20].

### Neglected Tropical Diseases

Neglected tropical diseases, while resulting in little mortality, cause substantial disability-adjusted life year (DALY) losses in developing countries [21]. Many of these diseases have a faeco-oral transmission pathway. Thus, improved sanitation could contribute significantly to a sustained reduction in the prevalence of many of them, including trachoma, soil-transmitted helminthiases, and schistosomiasis. Unfortunately, the current policy focus in most parts of the world is on treatment by medication, which, unlike good sanitation, is not a preferred solution because, in part, it is much more expensive.

Trachoma is endemic in many of the world's poorest countries. It is caused by the bacterium *Chlamydia trachomatis* and is the world's leading cause of preventable blindness [22]. Trachoma control is predominantly antibiotic-based despite the existence of the SAFE control strategy (surgery, antibiotics, face-washing, and environmental measures, namely sanitation promotion) [23],[24]. However, a recent cluster-randomised control trial in Ghana found that the provision of toilets reduced appreciably the number of *Musca sorbens* flies (the vector for trachoma) caught on children's eyes and by 30% the prevalence of trachoma, thus confirming the long-suspected role that sanitation could play in the control of trachoma [25].

Soil-transmitted helminths such as the large human roundworm, the human whipworm, and the human hookworms cause many millions of infections every year and many individuals are infected with more than one of these geohelminths [26]. Helminthic infections negatively impact the nutritional status of infected individuals, with consequent growth faltering in young children, and anaemia, particularly in pregnant women [27],[28]. Adult helminths live in the human gastrointestinal tract where they reproduce sexually. Their eggs are discharged in the faeces of the infected host and thus, mainly via open defecation, to other people. Ending the practice of open defecation with good sanitation can cut this transmission path completely, but most current helminth-control programmes focus on medication, which must be repeated periodically in the absence of sanitation [28],[29].

Globally, some 190 million people are infected with schistosomiasis, which can result in chronic debilitation, haematuria, impaired growth, bladder and colorectal cancers, and essential organ malfunction [28]. Adult schistosomes live in the portal veins where they pass their eggs into the environment via the urine (*Schistosoma haematobium*) or faeces (the other human schistosomes). After passing part of their life cycle in aquatic snails where they multiply asexually, cercariae are discharged into the water where they come into contact with and infect their human hosts through their skin. Thus, sanitation (and water) interventions are essential to any long-term control and elimination of schistosomiaisis, whereas the current standard intervention is repeated medication [29].

### Acute Respiratory Infections

With 4.2 million deaths each year (1.6 million among children under 5 years), acute respiratory infections are the leading cause of mortality in developing countries [30],[31]. Although sanitation is not directly linked to all acute respiratory infections, a recent study reported that 26% of acute lower respiratory infections among malnourished children in rural Ghana may have been due to recent episodes of diarrhoea [32]. Thus, sanitation could be a powerful intervention against acute respiratory infections.

### Undernutrition

Poor sanitation, hygiene, and water are responsible for about 50% of the consequences of childhood and maternal underweight, primarily through the synergy between diarrhoeal diseases and undernutrition, whereby exposure to one increases vulnerability to the other [33]–[35].

## Wider Benefits of Sanitation

In addition to its impact on health, improved sanitation generates both social and economic benefits. Householders understand these wider benefits [36] but scientists have only recently begun to study individuals' motivations for improving sanitation and changing sanitation behaviour.

While the main goal of agencies' sanitation programming is to improve health, householders rarely adopt and use toilets for health-related reasons. Instead, the main motivations for sanitation adoption and use include the desire for privacy and to avoid embarrassment, wanting to be modern, the desire for convenience and to avoid the discomforts or dangers of the bush (e.g., snakes, pests, rain), and wanting social acceptance or status [37],[38]. Furthermore, for women, the provision of household sanitation reduces the risk of rape and/or attack experienced when going to public latrines or the bush to defecate, and for girls, the provision of school sanitation facilities means that they are less likely to miss school by staying at home during menstruation [39].

The economic benefits of improved sanitation include lower health system costs, fewer days lost at work or at school through illness or through caring for an ill relative, and convenience time savings (time not spent queuing at shared sanitation facilities or walking for open defecation) (Table 1) [40].

**Table 1:** Economic benefits resulting from meeting the MDG sanitation target and from achieving universal sanitation access.

Population Benefitted and Economic Benefit	Meeting the MDGSanitation Target	Achieving UniversalSanitation Access
Population using improved sanitation (millions)	564	2,226
Diarrhoeal disease cases averted (millions per year)	190	673
Diarrhoeal disease deaths averted (thousands per year)	180	592
Health system costs saved ($ millions per year)	552	1,659
Patient non-medical costs saved ($ millions per year)	57	203
Value of lost working days avoided ($ millions per year)	1,056	4,010
Value of lives saved ($ millions per year)	1,718	7,294
Value of convenience time savings ($ millions per year)	31,320	149,923

Source: [40].

In total, the prevention of sanitation- and water-related diseases could save some $7 billion per year in health system costs; the value of deaths averted, based on discounted future earnings, adds another $3.6 billion per year [41]. Furthermore, in much of the developing world at any one time around half the hospital beds are occupied by people with diarrhoeal diseases [42]. Expressed at a national scale, poor sanitation and hygiene costs the Lao People's Democratic Republic 5.6% of its GDP per year [43] and studies in Ghana and Pakistan suggest that general improvements in environmental conditions could save 8%–9% of GDP annually [33].

Table 2 shows the cost–benefit ratios associated with achieving the Millennium Development Goal (MDG) sanitation target (a reduction of 50% in the proportion of people without improved sanitation by 2015 from the 1990 baseline figure) and with achieving universal sanitation access in the non-OECD (Organisation for Economic Co-operation and Development) countries. Thus, one dollar spent on sanitation could generate about ten dollars' worth of economic benefit, mainly by productive work time gained from not being ill if either of these goals were achieved.

**Table 2:** Cost-benefit ratios for achieving the MDG water supply and sanitation targets and for universal water supply and sanitation coverage.

Region	Cost–Benefit Ratio of Achieving the MDG Sanitation Target	Cost–Benefit Ratio of Achieving Universal Sanitation Access
Sub-Saharan Africa	6.6	6.5
Arab States	5.3	12.7
East Asia & Pacific	12.5	13.8
South Asia	6.9	6.8
Latin America & Caribbean	37.8	39.2
Eastern Europe & CIS	27.9	29.9
**Average for all non-OECD countries**	9.1	11.2

Source: [40].

Finally, the Disease Control Priorities Project recently found hygiene promotion to prevent diarrhoea to be the most cost-effective health intervention in the world at only $3.35 per DALY loss averted, with sanitation promotion following closely behind at just $11.15 per DALY loss averted [44].

## Analysis of the Current Situation

### Coverage

Currently, some 2.6 billion people lack access to improved sanitation, two-thirds of whom live in Asia and sub-Saharan Africa. 1.2 billion people, of whom more than half live in India, lack even an unimproved sanitation facility and must defecate in the open [4]. Regional disparities in sanitation coverage are huge. Whereas 99% of people living in industrialised countries have access to improved sanitation, in developing countries only 53% have such access. Within developing countries, urban sanitation coverage is 71% while rural coverage is 39%. Consequently, at present the majority of people lacking sanitation live in rural areas; this balance will shift rapidly as urbanisation increases. Worryingly, over the past two decades, provision of improved sanitation has barely kept pace with increasing populations while most other social services, including water supply, have outpaced population growth.

### Reasons for Slow Progress

For many years, national governments, aid agencies, and charities have subsidised sewerage and toilet construction as a means to improve access. This approach has resulted in slow progress for two main reasons. First, the programmes have tended to benefit the few relatively well-off people who can understand the system and capture the subsidies, rather than reach the more numerous poor people. Second, such programmes have built toilets that remain unused because they are technically or culturally inappropriate or because the householders have not been taught the benefits of them. In India, for example, many toilets are used as firewood stores or goat sheds [45],[46] and a recent study showed that about 50% of toilets built by a large government programme are not used for their intended purpose [47].

Even when appropriate toilets are promoted, their technical specifications frequently make them prohibitively expensive. Thus, a recent study in Cambodia found that while there is a strong demand for toilets, that demand remains mostly unrealised because people favour an unaffordable $150 design rather than simpler but still hygienic designs costing $5–$10 [48].

Another reason for slow progress is that disposal of children's faeces—the group most vulnerable to faeco-oral disease transmission—is neglected and under-researched. A recent literature review that analysed a wide range of disposal practices for children's faeces and the health gains that can result from them noted that this whole topic is significantly neglected [49].

Finally, sanitation is not an inherently attractive or photogenic subject. Before 2008, the International Year of Sanitation [50], sanitation specialists had failed to persuade politicians, the media, and other influential people of the importance of the subject. During 2008, however, there were many political events related to sanitation—notably regional sanitation conferences across the developing world—that resulted in Regional Sanitation Declarations, which have moved sanitation up the political agenda [51].

## Successful Approaches to Sanitation

Recently, there has been a shift away from centrally planned provision of infrastructure towards demand-led approaches that create and serve people's motivation to improve their own sanitation. Although sound technological judgment about appropriate solutions remains essential, appropriate programming approaches are now more important and contribute most to the success of sanitation work. Some of the most promising approaches that apply to both rural and urban sanitation are described below. Regarding the costs of these demand-led approaches, there are few published comparative studies, but sector professionals estimate that they cost less than traditional infrastructure provision. For example, the Water Supply and Sanitation Collaborative Council's Global Sanitation Fund allows average costs of $15 per person for demand-led approaches, whereas governmental provision of infrastructure typically costs tens to hundreds of dollars per person.

### Sanitation Marketing

Sanitation marketing uses a range of interventions to raise householders' demand for improved sanitation [38]. The approach involves understanding householders' motivations and constraints to sanitation adoption and use. These are then used to develop both demand- and supply-side interventions to ensure that appropriate sanitation products and services are available to match the demand. A successful example of sanitation marketing is described in Text S1.

### Community-Led Total Sanitation

Community-led total sanitation (CLTS) is a communications-based approach that aims to achieve “open defecation–free” status for whole communities rather than helping individual households to acquire toilets. CLTS was developed in Bangladesh (see section 2 in Text S1) and uses external facilitators and community volunteers to raise (“ignite”) community awareness that open defecation contaminates the environment and the water and food ingested by householders. It encourages a cooperative, participatory approach towards ending open defecation and creating a clean, healthy, and hygienic environment from which everyone benefits [52]. CLTS has spread from South Asia to Africa and South America in the past ten years and appears to be highly successful in certain communities. However, one recent study estimates that only 39% of ignited villages achieve open defecation–free status [53]. The success or failure of CLTS may relate to its cultural suitability and to the degree to which it addresses supply-side constraints to sanitation adoption [54].

### Community Health Clubs

Community Health Clubs aim to change sanitation and hygiene attitudes and behaviour through communal activities. The approach has proved effective and cost-effective in the Makoni and Tsholotsho Districts of Zimbabwe where villagers were invited to weekly sessions where one health topic was debated and then action plans formulated [55]. In one year in Makoni District, for example, 1,244 health sessions were held by 14 trainers, costing an average of US$0.21 per beneficiary and involving 11,450 club members. Club members' hygiene in both districts was significantly different (*p*<0.0001) from that of a control group, and the study's authors concluded that if a strong community structure is developed and the norms of a community are altered, sanitation and hygiene behaviour are likely to improve.

### Sanitation as a Business

Traditionally, sanitation has been regarded as a centrally provided service with little role for the creativity or energy of business. However, the increased demand created by sanitation marketing, CLTS, and Community Health Clubs can be met by the development of a vibrant local private sector for producing, marketing, and maintaining low-cost toilets [56]. For example, in Lesotho the national government organised and planned workshops for people to review toilet designs and building methods in its “local latrine builders” programme [57]. The local private sector can also be encouraged to become involved in pit-emptying, sale of safely composted human excreta as fertilizer, generation of methane from biogas toilets, and the operation of public toilets.

### Approaches Emphasising Low Cost

Many sanitation advocates now place the affordability of the toilets at the centre of the planning process. A common strategy is to encourage people to start with the simplest type of improved pit latrine (see section 3 in Text S1) and then to progress over time towards higher-specification and higher-cost toilets—the “sanitation ladder.” The critical and most cost-effective step on this ladder, for both health and social reasons, is the first step from open defecation to fixed-location defecation; the subsequent steps up the ladder may yield smaller incremental benefits.

### Approaches Specific to Urban Sanitation

Most successful demand-led approaches have been developed in rural contexts. Urban sanitation is much more complex, mainly because of higher population densities, less-coherent community structures, and the absence of opportunities for open defecation. Urban sanitation must extend beyond the household acquisition of a toilet to a systems-based approach that covers the removal, transport, and safe treatment or disposal of excreta (see section 4 in Text S1).

For on-site urban sanitation systems, pit-emptying services are common in middle-income countries where householders can afford the cost, but less common in poorer countries. However, in Maputo, Mozambique, a small community-based association has developed a pit emptying/septic tank desludging service using self-propelled machines to provide service in unplanned areas of the city [58]. For off-site or centralised systems, simplified or “condominial” sewerage systems, in which sewers are placed inside housing blocks and then discharged into conventional sewers if there are any nearby or led to a simple local wastewater treatment plant, can provide the same level of service as conventional sewerage but at around one-third to one-half of the cost [59].

In densely populated low-income urban areas, community-managed sanitation blocks, used only by community members who pay a monthly fee for operation and maintenance, are an option [60]. Public sanitation blocks that can be used by anyone, normally for a small fee per use, can be an acceptable alternative provided that they are well operated and maintained and have 24-hour access. Finally, in less densely populated low-income urban areas, on-site sanitation options of the types described in section 3 in Text S1 for rural areas are often applicable.

## The Role of the Health Sector in Improving Sanitation

Sanitation promotion is one of the most important roles the health sector can have in environmental health planning, because behaviours must be changed to increase householders' demand for and sustained use of sanitation, especially in rural areas where the pressure for change is lower. Thus, two of the most promising large-scale sanitation programmes in Africa are centred around demand creation and are both led and delivered by the Ministry of Health and its associated structures [37],[61],[62].

Sanitation can be promoted by the health sector through a stand-alone programme such as sanitation marketing or CLTS or included in disease-specific control programmes such as the ‘SAFE’ approach to trachoma [63]. Alternatively, it can be incorporated into a wider integrated community health package such as Ethiopia's HEP (Health Extension Programme), which was developed in 2004 to prevent the five most prevalent diseases in the country [61],[62]; safe sanitation and hygiene became a major focus within HEP because of the recognition that these diseases are all linked with poor environmental health.

Promotion alone by the health sector may be insufficient, however, to ensure sanitation adoption and maintenance. A “carrot and stick” approach may be needed in which sanitation coverage is increased through a combination of community-based promotion and enforcement of national or local legislation that every house must have a toilet [64],[65]. In many countries, Environmental Health Officers are responsible for ensuring the sanitary condition and hygienic emptying of toilets, and have the power to sanction dissenting households with fines and court action [65]. This enforcement role of the health sector is particularly important in urban areas where high-density living increases the risks of faecal contamination of the environment and where one person's lack of sanitation can affect the health of many other people.

The health sector also has an important role to play in advocacy and leadership. Politicians and the general public listen to doctors. That puts an onus on the medical profession to speak out on all important health issues, including sanitation. Historically, this has not happened. Thus, in 2008, *The Lancet* wrote, “the shamefully weak presence of the health sector in advocating for improved access to water and sanitation is incomprehensible and completely short-sighted” [66].

Given the huge potential health-cost savings achieved through improved sanitation, the health sector should be advocating for stronger institutional leadership, stronger national planning, and the establishment of clear responsibilities and budget lines for sanitation. Unfortunately, although the international health community puts large human and financial resources into many low- to medium-cost health interventions such as immunization and bed net distribution, it has been slow to act on the evidence showing that sanitation promotion and hygiene promotion are among the most cost-effective public health interventions available to developing countries.

Finally, the well-honed epidemiology and surveillance skills of health professionals must also now be applied to sanitation to establish clear links between national health information systems and sanitation planning and financing, which has historically been separate from health in most countries.

## Constraints to Success in Sanitation

The lack of national policies is a major constraint to success in sanitation (see section 5 in Text S1 for additional information on this and other constraints). Governments in general and health ministries in particular cannot play their key roles as facilitators and regulators of sanitation without policies that support the transformation of national institutions into lead institutions for sanitation, that increase focus on household behaviours and community action, that promote demand creation, and that enable health systems to incorporate sanitation and hygiene. Other constraints to success in sanitation are population growth and increasingly high population densities in urban and periurban areas of developing countries. Furthermore, most of the people who lack improved sanitation live on less than $2 per day, which makes high-cost, high-technology sanitation solutions inappropriate [44].

Finally, although macroeconomic analysis shows that sanitation generates economic benefit, the benefit does not necessarily accrue to the person who invests in the improved sanitation. So the economics at the household level remain a constraint to success in sanitation—many people are simply unable or unwilling to invest, given all the other competing demands on their money. This under-researched topic is currently under investigation by the WASHCost Project, which is studying the life-cycle costs of water, sanitation, and hygiene services in rural and periurban areas in four countries [67].

## Strategies to Achieve Success in Sanitation

Sanitation is a complex topic, with links to health and to social and economic development. It affects many but is championed by few. From our analysis of the situation, we believe that three major strategies could achieve success in sanitation.

The most important of these strategies is political leadership, which is manifested by establishing clear institutional responsibility and specific budget lines for sanitation, and by ensuring that public sector agencies working in health, in water resources, and in utility services work together better. The regional sanitation conference declarations [51] released during the International Year of Sanitation, in which many government ministers were personally involved, were an important step forward. In addition, the biennial global reports on sanitation and drinking water published by the World Health Organization and UNICEF [4],[68] contribute towards political leadership and aid effectiveness by publicising the sanitation work of both developing country governments and support agencies.

The second strategy is the shift from centralised supply-led infrastructure provision to decentralised, people-centred demand creation coupled with support to service providers to meet that demand. This strategy is transforming sanitation from a minor grant-based development sector into a major area of human economic activity and inherently addresses the problem of affordability, since people install whatever sanitation systems they can afford and subsequently upgrade them as economic circumstances permit.

The final strategy is the full involvement of the health sector in sanitation. The health sector has a powerful motivation for improving sanitation, and much strength to contribute to achieving this goal. The Declaration of Alma Ata in 1978 emphasised the importance of primary health care and included “an adequate supply of safe water and basic sanitation” as one of its eight key elements [69]. Many years have passed since this Declaration, and the body of evidence about sanitation has increased substantially. The health sector now needs to reassert its commitment and leadership to help achieve a world in which everybody has access to adequate sanitation.

## Supporting Information

Text S1 Supporting Information. Section 1, Sanitation Marketing in Benin; Section 2, Community-Led Total Sanitation in Bangladesh; Section 3, Sustainable Sanitation Technologies: Rural Areas; Section 4, Sustainable Sanitation Technologies: Low-Income Urban Areas; Section 5, Constraints to Achieving Success in Sanitation.
(0.59 MB PDF)

## Abbreviations

CLTS: community-led total sanitation
DALY: disability-adjusted life year
MDG: Millennium Development Goal
