# Supplementary material for: Sanitation and Health
Source: PLoS Med. 2010 Nov 16;7(11):e1000363. doi: 10.1371/journal.pmed.1000363 (PMC2981586; doi:10.1371/journal.pmed.1000363)
Supplement: Text S1 — Supporting Information. Section 1, Sanitation Marketing in Benin; Section 2, Community-Led Total Sanitation in Bangladesh; Section 3, Sustainable Sanitation Technologies: Rural Areas; Section 4, Sustainable Sanitation Technologies: Low-Income Urban Areas; Section 5, Constraints to Achieving Success in Sanitation. (0.59 MB PDF) [file pmed.1000363.s001.pdf]

SANITATION AND HEALTH

Duncan Mara, Jon Lane, Beth Scott & David Trouba

SUPPORTING INFORMATION

1. SANITATION MARKETING IN BENIN

In Benin, rural sanitation promotion is the responsibility of the Directorate for Hygiene and Basic Sanitation (DHAB) within the Ministry of Health. In the mid-1990s DHAB, with support from Danida and the World Bank, began testing a new approach to rural sanitation promotion using social marketing to motivate household demand coupled with toilet construction by local masons.

Over the years the approach has evolved into a successful rural sanitation programme which operates on three primary principles:

1. Zero hardware subsidy – households pay the full cost of toilet construction and maintenance themselves.
2. Use of marketing communications and a set of non-health related promotional messages (found to be the main motivations for toilet construction in rural Benin) to stimulate household demand for sanitation
3. Development of the capacity of small-scale private sector to provide quality construction services and offer a selection of low-cost toilet technologies.

The promotional messages focus on household-perceived benefits of sanitation – avoiding the threats of the bush (primarily snakes), exposure to weather (primarily rain) and the embarrassment of being seen defecating in the bush (privacy); preventing the contamination of food by flies attracted to faeces in the yard and the sense of pride afforded by having a household latrine. These messages are delivered via a number of different channels including local radio and community events and house-to-house visits conducted by village hygiene promotion volunteers.

DHAB trains two government-employed 'hygiene agents' per district whose responsibility is to train and monitor local masons in toilet design and construction, and two village hygiene promotion volunteers (usually one male, one female) per village (community of an average of 80-90 households) to carry out sanitation promotion, alongside the wider promotion of safe hygiene and water. The hygiene agents also provide household guidance on where to site a new toilet and check the quality of the toilet and certify it fit for use.

Data collected between 2006 and 2007, the most recent intervention round, show a 60% increase in the number of toilets constructed in 2007 compared to 2006. As DHAB only collects data on the toilets built within the intervention zones, this is likely to be an underestimate. DHAB is now scaling it up with a goal to reach 25,000 households across 64 areas within nine districts in Benin. Its vision and leadership has been critical to getting this far, while the Ministry of Health plays an enabling in promoting sanitation, developing private sector delivery capacity, quality assurance and monitoring and evaluation.[1]

Reference

1. Scott B, Jenkins MW, Kpinsoton M (2008) Changing sanitation behaviour in Africa. Paper presented at AfricaSan 2008, Durban, South Africa, 18−21 February.

2. COMMUNITY-LED TOTAL SANITATION IN BANGLADESH

In 2000, the development consultant Kamal Kar, an expert in participatory approaches, introduced Community-Led Total Sanitation (CLTS) in Mosmoil, a village in Rajshahi district, Bangladesh. This bottom-up approach spread fast in Bangladesh, where NGOs, such as VERC (Village Education Resource Centre), informal institutions and international NGOs, such as Plan International and the Water and Sanitation Programme, supported its implementation.[1] The approach was embraced by the

Government, which in 2005 set a target of achieving 100% sanitation by 2010. Coverage is reported to have increased from 33% in 2003 to 70% in 2006, with particular success in the rural areas but less progress in the poor urban slums and settlements.

A 2008 paper by Howes et al. examined the results of the work by three different organizations: VERC, in Naogaon; Dishari, in Lalmonirhat; and CARE, in Rangpur.[2] The CLTS village sites varied socio-economically, geographically and culturally, and the organizations therefore worked in different ways and parts of the country.

After the introduction of CLTS, some open defecation continued (household field studies began in 2007, about 2 or 3 years after CLTS 'ignition'), but in general large numbers of rural people were persuaded to adopt and use sanitary latrines through CLTS. This was considered a success since the great majority of households in the selected communities had had no latrine and used to defecate in the open, which contributed to high levels of diarrhoea, especially among children.

References

1. http://www.clts-global.org/page/clts-approach1.
2. Howes M, Enamul H, and Abu N (2009) Community-led sanitation and its successors in Bangladesh. London: Institute of Development Studies.

3. SUSTAINABLE SANITATION TECHNOLOGIES: RURAL AREAS

There are several definitions of 'sustainable sanitation'. Mara defines it as "a sanitation system that is affordable; socially, technically, physically and institutionally feasible; able to be used easily, properly and on demand, and able to be maintained easily, regularly and at low cost, by its users, including women and children, in the long term; provides a hand-washing facility (or has one nearby); and has no adverse effects on the environment".[1]

In dispersed rural areas often the most suitable sanitation system is the Arborloo (Figure S1).[2] This is a shallow pit latrine (typically 0.8 m in diameter and 1 m deep) which is used for 6−12 months; after each use soil, ash or leaves are added to the pit. When it is full to within around 20 cm of the ground surface the latrine superstructure is placed over a new pit, and the full pit covered with soil and a fruit or medicinal tree planted in it. Eventually the household has an orchard of high-value trees which generates both high-quality fruit and an income from the sale of excess fruit and/or medicinal products. In many rural households the men are away working in nearby towns or mines and the women like the Arborloo as they can dig the shallow pits quite easily; moreover there is the advantage that, while the nutrients in the excreta are used to promote the growth of the trees, there is no handling of either faeces or urine.

Alternatives to the Arborloo include urine-diverting alternating twin-vault ventilated improved vault latrines (UD-VIVs), ventilated improved pit latrines (VIPs), and pour-flush toilets (PFs); they are all also suitable in higher-density rural areas and in low-to-medium density urban areas.[3] Of these the currently most promising is the UD-VIV.

UD-VIVs are wholly above ground and comprise two separately ventilated vaults which receive only faeces and anal cleansing materials; the urine, diverted in a specially designed toilet bowl or squat-plate, is discharged into a small adjacent soakaway − this is done to keep the vault contents from becoming too wet, so they can dehydrate easily (Figure S2).[4] One vault is used for 12 months, when the other vault is put into service; after the second vault has been used for ~11 months the first vault is emptied by the householder (using a long-handled shovel; the vault contents are buried on site), so that the first vault can be put back into service at the beginning of the third year − this sequence of alternating vault usage continues indefinitely.

Ecological sanitation ('EcoSan') systems, which have been the subject of much research and development, are based on the principle that one person's excreta contain almost all the nutrients required to produce that person's basic carbohydrate crop (potatoes, rice, maize, for example). Since most of the nutrients are in urine, it makes sense to separate urine ('yellow water') and faeces plus any

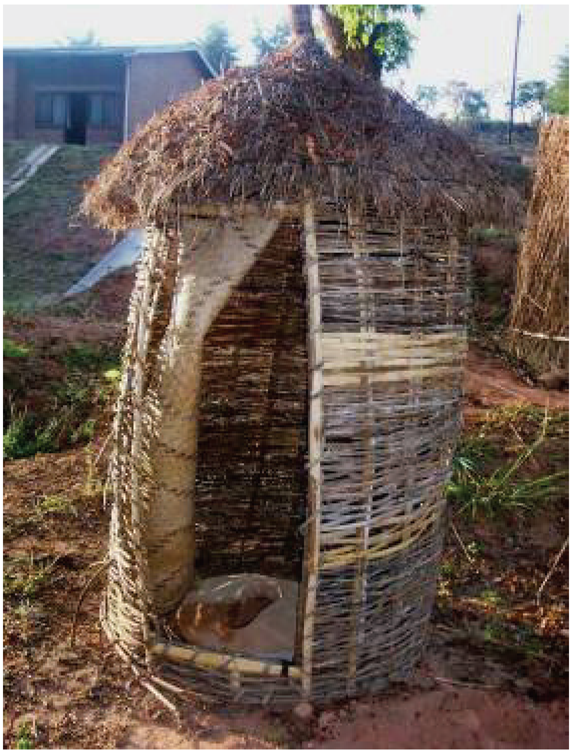
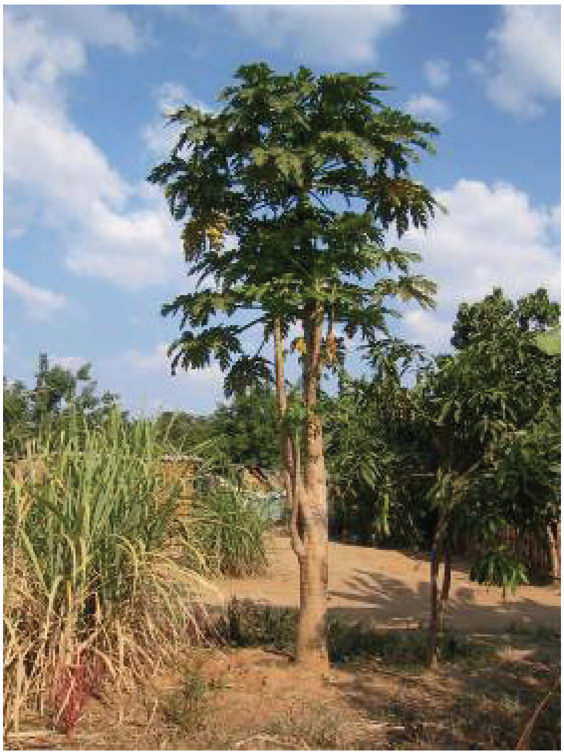

Figure S1. Left, an Arborloo; right, a papaya tree planted in an Arborloo pit.

flush water ('brown water') and not to mix either of them with 'grey water' (all the non-toilet wastewater).[5] Arborloos can be considered a very simple form of EcoSan. The principal problem with EcoSan systems is their high costs. However, UD-VIVs can easily be converted to EcoSan systems by not discharging the urine to a soakaway, but using it after dilution with water or grey water for vegetable or crop irrigation, and by not locally burying the vault contents, but using them as a soil conditioner and so aiding vegetable or crop production.

More detailed descriptions of all these sanitation systems are available online.[6]

References

1. http://duncanmarasanitation.blogspot.com/2008/08/what-is-sustainable-sanitation.html.
2. http://www.personal.leeds.ac.uk/~cen6ddm/Arborloos.html.
3. http://www.personal.leeds.ac.uk/~cen6ddm/WatSan.html.
4. http://www.personal.leeds.ac.uk/~cen6ddm/eThekwiniLatrines.html.
5. http://www.personal.leeds.ac.uk/~cen6ddm/EcoSan.html.
6. http://www.personal.leeds.ac.uk/~cen6ddm/SanitationPractice.html.

4. SUSTAINABLE SANITATION TECHNOLOGIES: LOW-INCOME URBAN AREAS

In low-income high-density urban areas there is seldom space for on-site sanitation systems, and the choice is normally between simplified sewerage, low-cost combined sewerage, and community-managed or public sanitation blocks.

Simplified or 'condominial' sewerage was developed in Northeast Brazil in the 1980s to provide the same level of service as conventional sewerage but at around one-third to one-half of its cost.[1] Rigorous hydraulic design (more rigorous, in fact, than that used for conventional sewerage) results in a 100-mm diameter sewer laid at a gradient of 1 in 200 being able to serve ~1200 people with a water consumption of 100 litres per person per day (or, for example, ~2400 people with a water consumption of 50 litres per person per day). The sewers are placed inside the housing block (rather

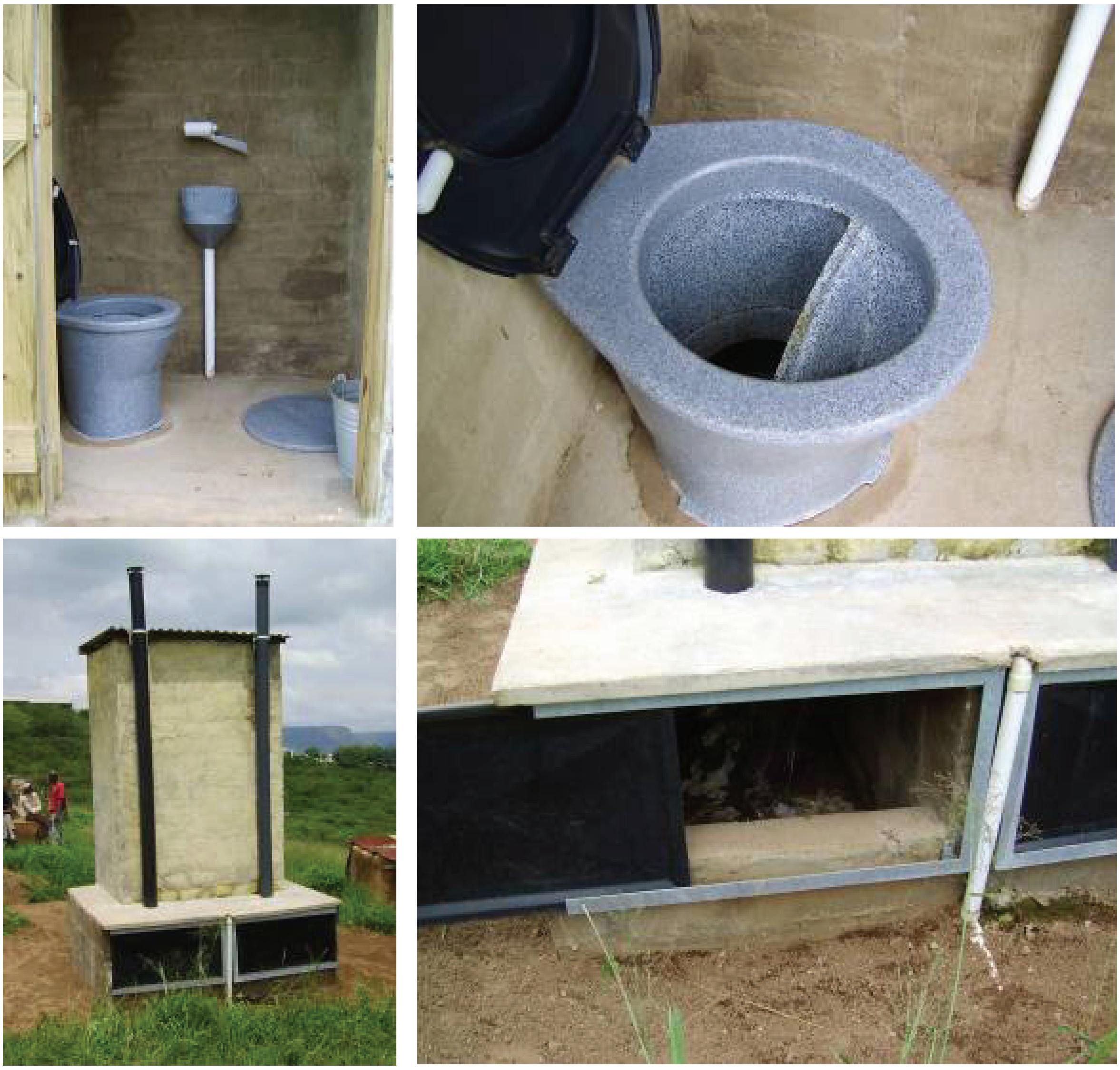

Figure S2. A UD-VIV latrine − top left: front view showing the urine-diverting pedestal seat unit, urinal, and cover over the entrance to the vault not in use; top right: close-up of the urine-diverting pedestal seat unit; bottom left: rear view showing the two vent pipes (one for each vault) and the small urine pipe (in the centre) which leads to an adjacent soakaway; and bottom right: close-up of one of the two vaults with the sliding door open showing the dehydrating vault contents.

than in the middle of the road) and very simple sewer junction and inspection boxes are used (rather than much more expensive manholes). The monthly charges for simplified sewerage are consequently low − for example, in Northeast Brazil in January 2008 they were USD 3.50 per household per month (only 1.7 percent of the minimum wage). In northeast Brazil simplified sewerage is cheaper than on-site sanitation systems at population densities above ~160 persons per hectare. The simplified sewers discharge into conventional sewers if there are any nearby, or lead to a simple wastewater treatment plant such as waste stabilization ponds.[2]

In low-income coastal areas subject to regular (annual or biannual) flooding low-cost combined sewerage, as developed in low-income settlements in the state of Rio de Janeiro, Brazil is often cheaper than simplified sewerage and separate stormwater drainage.[3] As with simplified sewerage, wastewater treatment is required.

In very low-income areas where simplified and low-cost combined sewerage are unaffordable one promising solution is to have community-managed sanitation blocks such as those developed by the Indian NGO SPARC (the Society for the Promotion of Area Resource Centres).[4] These normally

comprise separate toilet cubicles for men and women, handwashing facilities, showers and a laundry area, together with a community meeting room and kitchen. The excreta are often digested anaerobically in a septic tank or equivalent beneath the toilet compartments, and the resulting biogas collected and used on-site for cooking. Such blocks are open to the members of that community only, so are defined as shared but not public toilets. There are various public, private or civil society organisations managing public toilet blocks around the world; some do this very well (e.g., Sulabh International[4]), but often the main drawback is maintaining them in a hygienic condition in the long-term.

More detailed descriptions of all these sanitation systems are available online.[5]

References

1. http://www.personal.leeds.ac.uk/~cen6ddm/simpsew.html.
2. http://www.personal.leeds.ac.uk/~cen6ddm/WSP.html.
3. http://www.personal.leeds.ac.uk/~cen6ddm/LowCostCombinedSewerage.html.
4. http://www.personal.leeds.ac.uk/~cen6ddm/CommunalSanitation.html.
5. http://www.personal.leeds.ac.uk/~cen6ddm/SanitationPractice.html.

5. CONSTRAINTS TO ACHIEVING SUCCESS IN SANITATION

Some 2.5 billion people lack access to improved sanitation, two-thirds of whom are in Asia and sub-Saharan Africa, and over one billion people defecate in the open. The health implications are huge. Diarrhoeal diseases claim nearly 2 million lives of children under 5 per year. Population growth, mostly in urban and periurban areas of developing countries, jeopardizes efforts to improve sanitation coverage and control disease, as does poverty. Most of the people lacking improved sanitation live on less than $2 per day, which makes high-cost, high-technology sanitation solutions inappropriate.

There is no single constraint or challenge underlying the sanitation gap; governance, awareness, technical, management, and financing barriers all play a role. In plain terms this means aspects such as transforming governmental institutions; focusing on household behaviours and community action; promoting sanitation and hygiene in order to increase demand; improving the effectiveness of subsidies, where they exist; sanitation and hygiene; and spending money more wisely.

Perhaps the greatest challenge involves politicians at the national level, who often fail to understand the guiding principles behind sanitation. Sanitation work is unglamorous, difficult and combines social sciences, political, institutional and technical work. It is slow steady work, house by house, community by community. In this sense, it is different from drinking water provision, and the health benefits are not as immediately obvious. Compared to sharing facilities for an intimate experience like going to the bathroom, people are less averse to sharing a tap which provides good quality water. Politicians who make decisions on sanitation also face other entrenched interests and powerful ministries (health, finance, defence, agriculture, etc.) which compete for financing and importance.

Another constraint is the level of understanding by people themselves at the household level. When people do value sanitation, and thus demand it, it is often for reasons other than health. These reasons – privacy, status, cleanliness, etc. – are not unimportant, but the connection between improved hygiene and health is often poorly understood. Marketing sanitation and promoting behaviour change are key areas where most countries have few skills, few incentives and limited capacity.

Another stumbling block is the limited knowledge and awareness about appropriate sanitation systems and technologies that keep project costs affordable and acceptable, while providing people with a fully acceptable level of service. This is primarily a constraint within the sector on the part of professionals. However, while the sanitation sector is increasingly aware of the need to design programmes that are both socially and environmentally appropriate and sustainable, promoters of household-centred environmental sanitation and similar planning approaches still face a number of misconceptions such as the myth that basic and simple technology cannot be good technology. From single pit to semi-centralized sanitation, such systems and technologies are in fact quite diverse and adaptable for the cultural and physical conditions where they need to be implemented (see sections S3 and S4 above).

The health sector's lack of engagement in sanitation is also a constraint, a motivating factor for the publication of this series by *PloS Medicine*. It has been shown that proper toilets dramatically reduce diarrhoea, cholera, pneumonia, worms, and malnutrition.

While money − or the lack of it − is often cited as a constraint, in fact, most funding for sanitation comes from a single source, the household. Households contribute directly through investments in latrines, soap and home improvements, and by paying local levies for sanitation services. They also pay indirectly through taxes which pay for public services and repayment of debt. The money that does come into sanitation work from international donors, aid agencies and national governments might be more wisely spent in support of existing national or local programmes on awareness building, health education and hygiene promotion, rather than subsidized toilet construction. Substituting appropriate levels of household investment for public investment may free up additional funds from sources such as the central government, regional, local and urban governments, donors, and others.

National policies – or the lack thereof − are also a constraint because they have not enabled governments to play their key roles as a facilitator and regulator of sanitation. Governments have been unable or unwilling to foster and promote innovation while holding service providers accountable and affording the right degree of protection to the environment. A fundamental starting point is for governments and support agencies to opt for policies which support demand-led approaches. Behaviour-change based models such as Community-led Total Sanitation (and other approaches) appear to be at least as effective if not more so at delivering services and facilitating behaviour changes.

Even more than water, sanitation suffers from a combination of institutional fragmentation, weak national planning and low political status. Governments need to allocate responsibility for sanitation to a single ministry within the country. This is because sanitation needs clear leadership in order to reflect the importance of sanitation to economic and social progress within the country. It also helps in coordinating with other stakeholders working in poverty, hunger, education and gender equality – where MDG targets also exist and whose achievement depends much on improved sanitation.

Separate budget lines for sanitation will improve the ability of governments to monitor and evaluate the effectiveness of spending in the sector, particularly as it relates to health gains through improved sanitation. Disaggregating spending on sanitation will help generate more knowledge on what works and what does not work in the sector, and it will help sanitation professionals better define what type of funding, and how much, is needed to achieve successful and sustainable sanitation programmes.

****
